# Simpson's paradox visualized: The example of the Rosiglitazone meta-analysis

**DOI:** 10.1186/1471-2288-8-34

**Published:** 2008-05-30

**Authors:** Gerta Rücker, Martin Schumacher

**Affiliations:** 1 Institute of Medical Biometry and Medical Informatics, University Medical Center Freiburg, Germany

## Abstract

**Background:**

Simpson's paradox is sometimes referred to in the areas of epidemiology and clinical research. It can also be found in meta-analysis of randomized clinical trials. However, though readers are able to recalculate examples from hypothetical as well as real data, they may have problems to easily figure where it emerges from.

**Method:**

First, two kinds of plots are proposed to illustrate the phenomenon graphically, a scatter plot and a line graph. Subsequently, these can be overlaid, resulting in a overlay plot. The plots are applied to the recent large meta-analysis of adverse effects of rosiglitazone on myocardial infarction and to an example from the literature. A large set of meta-analyses is screened for further examples.

**Results:**

As noted earlier by others, occurrence of Simpson's paradox in the meta-analytic setting, if present, is associated with imbalance of treatment arm size. This is well illustrated by the proposed plots. The rosiglitazone meta-analysis shows an effect reversion if all trials are pooled. In a sample of 157 meta-analyses, nine showed an effect reversion after pooling, though non-significant in all cases.

**Conclusion:**

The plots give insight on how the imbalance of trial arm size works as a confounder, thus producing Simpson's paradox. Readers can see why meta-analytic methods must be used and what is wrong with simple pooling.

## Background

Simpson's paradox, also known as the ecological effect, was first described by Yule in 1903 [1] and is named after Simpson's article in 1951 [2]. It refers to the phenomenon that sometimes an association between two dichotomous variables is similar within subgroups of a population, say females and males, but changes its sign if the individuals of the subgroups are pooled without stratification. This is reflected in the title of a paper by Baker and Kramer ('Good for women, good for men, bad for people', [3]). There are numerous examples, particularly from the areas of epidemiology and social sciences, of associations strongly affected by observed or unobserved dichotomous variables [4-8]. Even a tale based on Simpson's paradox has been told [9]. The reason for its occurrence is the existence of an influencing variable that is not accounted for, often unobserved. Thus, it may seem that the effect is charactistic for observational studies and can be avoided by randomization.

This is not true, as was pointed out by others [10-14]. As Altman and Deeks note, Simpson's paradox is not really a paradoxon, but a form of bias, resulting from heterogeneity in the data if not accounted for [10]. Often tables of hypothetical as well as real data examples are presented. However, though these examples are easily recalculated, there is a need for readers, especially clinicians and practitioners in other fields, to really understand the nature of the phenomenon.

Baker and Kramer proposed a plot, later called the Baker-Kramer (BK) plot, which was independently invented by others much earlier, for graphically illustrating Simpson's paradoxon [3,13-15]. Their examples stem from hypothetical data. For this plot it is required that the influencing variable is dichotomous. In the setting of a meta-analysis, however, the main source of heterogeneity and thus the most important influential variable is well-known and not dichotomous in general: it is the variable 'trial'. A perfect example of Simpson's paradox occurring in a meta-analysis of case-control studies is given by Hanley and Theriault [8]. In this meta-analysis all single trials show an increased risk for exposed individuals, while the pooled analysis reverses this effect.

As a (less perfect) example for meta-analysis of RCTs, we use a recent systematic review of the effect of rosiglitazone on the risk of myocardial infarction and death from cardiovascular diseases [16]. It stated a significant increase of myocardial infarctions in the rosiglitazone group. The authors found a Peto odds ratio 1.428 with 95 per cent confidence interval [1.031; 1.979] and p-value 0.0321 (fixed effect model) [17]. This meta-analysis immediately raised a discussion not only about the safety of the drug, but also on methodological issues referring to potential heterogeneity, different follow-up times, the large number of trials with no or very few events and the imbalanced group sizes within many trials [18-20]. A re-analysis of the data using several variants of the Mantel-Haenszel method found that the significance of the effect is questionable (odds ratio estimates between 1.26 and 1.36, most of them not significant) [18]. Though not consistently significant, meta-analysis (all methods) exhibits an excess of events in the treatment group (rosiglitazone), compared to the control group (any other regimen). For example, taking the risk difference (fixed effect model, Mantel-Haenszel method) results in a combined estimate of 0.002 (95 per cent confidence interval [0.000; 0.004] with p-value 0.0549), corresponding to an estimated NNH (Number Needed to Harm) of about 489 patients.

One problem of this data is the large number of trials without any events. If the outcome is measured by the risk ratio or the odds ratio, these trials are often excluded from a meta-analysis because it is argued that they do not contribute any information about the magnitude of the treatment effect [21]. In order to use all available information, simple pooling of all single tables could be rather tempting. It is seemingly convenient here because of the considerable number of double-zero studies, despite of the general consensus that this is discouraged [22]. If pooling is done – in spite of this objection – for the main endpoint myocardial infarction (MI), we in fact surprisingly observe that the pooled 2 × 2-table provides the contrary: the risk of MI for the treated individuals is 0.0055 and therefore less than for the control group (0.0059), see Table 1. The pooled odds ratio is 0.94 with 95 per cent confidence interval [0.69; 1.29] (p-value 0.7109). This (non-significant) effect reversion, produced by pooling, was observed by another author who in the light of these found the results of the meta-analysis 'intriguing' [23]. It can be seen as a milder form of Simpson's paradox.

**Table 1:** Pooled data of rosiglitazone meta-analysis (full data see ref. [16])

	Events	Total	Fraction
Rosiglitazone group	86	15556	0.5528%
Control group	72	12277	0.5865%

In the next section, we first develop two kinds of plots to reveal and illustrate the mechanism of Simpson's paradox and effect reversion, using the rosiglitazone example. The third plot emerges from overlaying both plots. In the results section, we apply the plots to the data given by Hanley and Theriault [8] and discuss both methods and results. The paper is ended with conclusions.

## Methods and Results

### Simpson's paradox for continuous variables

**Figure 1:**
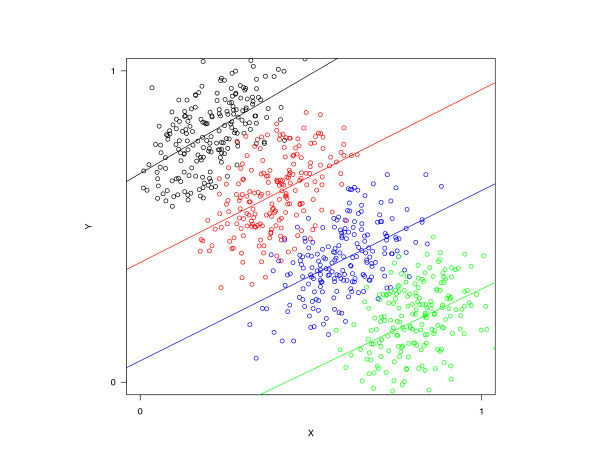
**Scatterplot of correlation between two continuous variables *X *and *Y*, grouped by a nominal variable *Z*.** Different colors represent different levels of *Z*.

The first idea to give a pictorial representation of the data is very simple. It comes from a graphic that serves for demonstrating the continuous version of the effect. For example, think of a correlation study where the data are grouped by a nominal variable *Z*, say study center. The conditional correlation (i.e. the correlation, given *Z*) of two continuous variables *X*, *Y *is assumed to be positive for all values of *Z*. Simpson's paradox occurs if, on the other hand, *between *different levels of *Z *holds 'the higher *X*, the lower is *Y *'. The appropriate plot best illustrating this is given by Figure 1. It is a grouped scatterplot that shows approximately parallel ascending regression lines within each level of *Z*, but a decreasing sequence of midpoints. Our goal is now to transfer this idea to the case of both *X *and *Y *being dichotomous.

### Simpson's paradox for dichotomous variables: a scatterplot

Let *X*, *Y *be dichotomous variables, where *X *is the treatment (1 = active, 0 = control) and *Y *is the outcome of interest (e.g., 1 = MI, 0 = no MI, where MI means myocardial infarction). The grouping variable is denoted as *Z*. In our meta-analytic example, *Z *∈ {1,..., *N*} is the trial (*N *= 42 for the rosiglitazone meta-analysis). Simpson's paradox occurs, e.g., if *within *(most) studies, the event *Y *is more frequent in the active treatment group (*X *= 1), but *between *studies, those with larger treatment proportions (corresponding to higher *X*) tend to exhibit *lower *event probabilities (corresponding to lower *Y*). This is possible only if the proportions of patients treated with the active drug vary substantially over all trials. Exactly this – the noticeable imbalance of the groups in many of the studies – is a characteristic feature of the rosiglitazone meta-analysis, as is pointed out both in the original article [16] and several reactions thereon, e.g. [18]. The connection between group imbalance and the occurrence of ecological effects was pointed out earlier by some authors [8,10,11].

**Figure 2:**
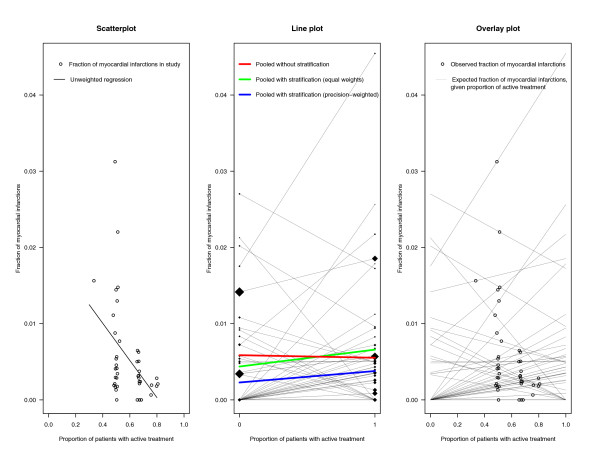
**Three plots elucidating effect reversion in rosiglitazone meta-analysis: (a) Scatterplot of fraction of events against proportion of patients in the active treatment group (left panel). **(b) Line plot displaying risk differences within trials (middle panel). 0 = control group, 1 = active treatment group. (c) Overlay plot of scatterplot and line plot (right panel).

Figure 2 (left panel) is a straightforward analogue to the continuous plot described above. Instead of the (dichotomous) variables *X *and *Y *themselves, their observed frequencies are used. A simple scatterplot is presented that shows the overall event frequencies *P*(*Y *= 1|*Z *= *i*) within the *N *trials *i *= 1,..., *N *versus the proportions *P*(*X *= 1|*Z *= *i*) of patients undergoing the active treatment. The large dispersion of the treatment proportions, unusual for randomized trials, is clearly seen. The negative correlation between treatment proportion and event probability (indicated by the fitted unweighted regression line) could lead to the deceptive impression that the frequency of adverse events decreases if more patients receive active treatment, thus potentially producing Simpson's paradox.

### Simpson's paradox for dichotomous variables: a line plot

A second way to demonstrate this is given by Figure 2 (middle panel). It shows, according to the scatterplot for continuous variables, the actual treatment *X *(i.e, 0 or 1) on the *x*-axis and the event frequencies conditional on group (*X *= 0 or *X *= 1) and trial (*Z*), that is *P*(*Y *= 1|*X*, *Z*) on the *y*-axis. Points belonging to the same trial are joined by a thin line, so that different lines indicate different trials. The slope of each *within-trial line *corresponds to the risk difference of this trial. The lines show a tendency to increase for most trials, revealing more adverse events in the active treatment group, in agreement with the published result of the meta-analysis.

In addition, three other lines are drawn. The green line joins the estimated mean event frequencies under control and under rosiglitazone, calculated within trials and averaged with equal weights for all trials. The blue line is similar, but the trials are now weighted with their precision, measured by the inverse sampling variance, calculated from a meta-analysis using the risk difference as outcome measure. Both lines increase slightly, reflecting what in average happens within trials.

The red line, however, calculated by simple collapsing all 2 × 2-tables without stratification by trial, *de*creases. The reason is that there are many unbalanced trials with the treatment groups being larger than the control groups and simultaneously having the lowest event rates (see Figure 2, left panel). We can visualize this by adding further elements to this plot. The starting and ending points of the single trial lines are marked by diamonds with size proportional to the size of the control group and the treatment group of this trial, respectively. If this is done, the contribution of single trial arms to the red line becomes visible. In our example, we have a large trial with many events in the control group (left) and, on the other hand, many trials with a larger proportion of rosiglitazone patients having low event rates (right-hand side).

### Simpson's paradox for dichotomous variables: the overlay plot

The right panel plot of Figure 2 shows a combination of the scatterplot and the line plot. The circles from the scatterplot and the trial-specific lines from the line plot are overlaid, while the regression line, the colored lines and the diamonds are skipped for sake of clarity. The interpretation of the *x*-axis and the lines now is slightly changed. Values *x *on the *x*-axis are interpreted as all possible proportions of active treatment in a trial. The *y*-values on the line belonging to a particular trial indicate the expected frequency of events in this trial, given *X *= *x*. If *X *= 0, this corresponds to the observed fraction of events in the control group (the intercept). If *X *= 1, the value provides the observed fraction of events in the treatment group of the trial. The *i*'th line is thus given by the linear equation

(1) *y *= *P*_*Z *= *i*_(*Y *= 1|*X *= 0) + [*P*_*Z *= *i*_(*Y *= 1|*X *= 1) - *P*_*Z *= *i*_(*Y *= 1|*X *= 0)] *x*,

where the slope *P*_*Z *= *i*_(*Y *= 1|*X *= 1) - *P*_*Z *= *i*_(*Y *= 1|*X *= 0) is the risk difference observed in trial *Z *= *i*, as stated above. If we insert for *x *the proportion *x*_0 _of patients actually treated in trial *i*, that is *x*_0 _= *P*_*Z *= *i*_(*X *= 1), we get

*y*_0 _= *P*_*Z *= *i*_(*Y *= 1|*X *= 0) + [*P*_*Z *= *i*_(*Y *= 1|*X *= 1) - *P*_*Z *= *i*_(*Y *= 1|*X *= 0)] *P*_*Z *= *i*_(*X *= 1),

which results (after straightforward simplification) in *y*_0 _= *P*_*Z *= *i*_(*Y *= 1), the overall frequency of events in trial *i*. These values are marked as the circles on the lines in the right-hand panel, which are the same as those on the scatterplot (left panel). This equality corresponds to equation (1) in [4].

### Application

**Figure 3:**
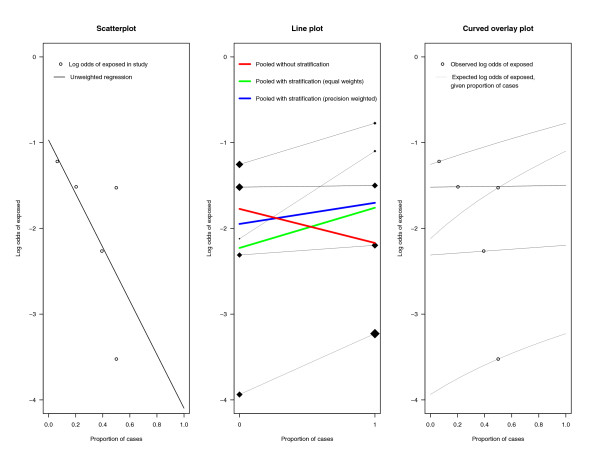
**Three plots illustrating Simpson's paradox in a meta-analysis of case-control studies: (a) Scatterplot of frequency of exposition (on a log odds scale) against proportion of cases (left panel).** (b) Line plot displaying log odds ratios within studies (middle panel). 0 = control group, 1 = case group. (c) Curved overlay plot (right panel).

We apply the plots to the example of meta-analysis of case-control studies given by Hanley and Theriault (data in reference [8]). The cases are children with leukemia, the exposition of interest being the presence of a high voltage power line within 100 m of the residence. Figure 3 displays the plots for this example. The *y*-axes are logit-transformed, because effect is measured as odds ratio. The scatterplot (left-hand panel) shows that the proportion of exposed (children living near a power line, here expressed as log odds) was higher in studies with a lower case-control ratio. The line plot (middle panel) displays that within all studies the exposition is slightly associated with leukemia, likewise for the stratified meta-analysis (green and blue line), but in the pooled sample (red line) the direction of association is reversed. The diamonds disclose how the large case and control groups pull the red line in the opposite direction. A direct overlay of these plots would not make sense, because when using a nonlinear transformation of the *y*-axis, the circles of the scatterplot do not lie exactly on the lines of the line plot. Instead, by subjecting equation (1) to the logit transformation we get a curved counterpart to the overlay plot. This is shown in the right-hand panel of Figure 3.

## Discussion

The example of the rosiglitazone meta-analysis illustrates that an ecological effect can occur even if all studies are randomized clinical trials. The scatterplot, applied to this example, shows that the myocardial infarction rate is the lower, the higher the proportion of patients in the active treatment groups is. This is no effect of the treatment, but an artefact of the studies included in this meta-analysis. The large majority of treated patients in some trials is explained by the fact that the authors pooled multiple groups of patients receiving rosiglitazone, where applicable [16]. On the other hand, many of these studies had only short-time follow-up, so that there were only few events observed. Casually, we note that this kind of heterogeneity in study design is present although there was no indication of statistical heterogeneity of the treatment effect on any scale, as measured in terms of *τ*^2^, *H *or *I*^2^[24]. These measures do not capture heterogeneity in other respect. This taken into account, the result of the meta-analysis that more adverse events are attributed to treatment than to control, as claimed to be significant in [16], was questioned by others [18].

In general, even a strong correlation contrary to the within-study association does not necessarily cause an effect reversion. This happens only if the disparities of the treatment arm sizes are large enough to outbalance the treatment effect in the single trials. This can be judged by inspection of the line plot. The line plot displays the treatment effect in each single study, as the slope of each line corresponds to the treatment effect measured in this study. The slope of the green line is the (uniformly weighted) mean treatment effect, that of the blue line the weighted mean treatment effect, the latter corresponding to the result of a meta-analysis. This kind of plot is not restricted to the risk difference, as the second example shows. Rather, it is easily generalized to a plot for the risk ratio or the odds ratio or other measures of treatment effect, such as the arcsine difference [25], by using the log scale, the logit scale, or the arcsine scale for the *y*-axis, respectively.

If the *y*-axis is not transformed, the plots can be overlaid. At first glance, the overlay plot is evocative of the so-called BK-plot [3,13-15]. It was first demonstrated using a hypothetical situation with only two groups (males and females), with the female fraction of patients as *x*-axis and the two lines corresponding to the two treatments [3]. The BK-plot was applied, for example, to medical school admission data [26]. There is, however, a fundamental difference between our overlay plot and the BK-plot which is elucidated in Table 2. In the overlay plot, the *x*-axis represents the variable 'treatment', that is, proportions of patients treated with the active treatment, and the lines correspond to any number of strata (here trials). In the BK-plot, however, *x *represents a binary confounder (i.e., proportions of patients belonging to one of two subgroups), and the lines correspond to the treatments. In fact, the BK-plot was originally introduced to elucidate Simpson's paradox for the simplest case that both the treatment variable and the confounding variable are binary. The plot then contains only two lines and two circles. Insight comes from comparing the position of the two circles on these lines: If Simpson's paradox is working, the lines have the same direction, do not intersect, and the circle on the lower line lies higher than the circle on the upper line. This method of pairwise comparing circles does not work in the context of a large and maybe heterogeneous meta-analysis. The confounder, here the trial, is not binary. Moreover, Simpson's paradox in meta-analysis uses to occur in a generalized form: We do not presuppose that the effects within all studies have the same direction. An effect reversion is identified if the sign of the pooled effect differs from that of the within-study treatment effect, estimated using meta-analytic methods.

**Table 2:** Overlay plot compared to Baker-Kramer plot [3]

Element of the plot	Plot type	
	Overlay plot	Baker-Kramer plot

*x*-axis	Treatment (proportion)	Binary confounder (proportion)
*y*-axis	Outcome	Outcome
Lines	Strata (here: Trials)	Treatments

As mentioned before, looking at the scatterplot or the overlay plot alone does not suffice, because a strong association between the proportion of patients treated and the event frequency in the direction opposite to the treatment effect is not sufficient for an effect reversion. The essential information is given by the line plot or by using the whole triplet of plots.

In addition, we screened a large set meta-analyses for finding further examples of this phenomenon. This data set, consisting of 157 meta-analyses with binary endpoints and two treatment groups was kindly provided by Peter Jüni who had collected the data at the Department of Social and Preventive Medicine, University of Berne, Switzerland. We had formerly used these data for a study on publication bias [27]. For each meta-analysis, a 'Simpson check' is carried out by comparing the sign of the result of the pooled analysis to the sign of the meta-analytic result, using the risk difference (without loss of generality). We found that in 9 out of all 157 meta-analyses (5.7%) the sign changed. However, in all these examples the treatment effect was far from being significant, and the confidence intervals of the meta-analytic and the pooled estimate overlapped largely. Hence the change of the sign was of no statistical importance.

## Conclusion

The rosiglitazone example illustrates that an ecological effect (Simpson's paradox) can occur even when all studies are randomized clinical trials. However, as our empirical study shows, this is not a common phenomenon. When it occurs, it is caused by strong imbalance of the proportions allocated to the active and control treatment in the trials included in the meta-analysis. The usual measures of heterogeneity on the treatment effect scale are not sensitive against this kind of heterogeneity.

In our opinion, the plots proposed here serve to clarify what is going on beyond the calculations. Taken together, they help the reader to understand what is behind Simpson's paradox if he faces it in a meta-analysis. The R code producing the plots is available from the first author on request [28].

## Competing interests

The authors declare that they have no competing interests.

## Authors' contributions

GR conceived the proposed plots and drafted the manuscript. MS contributed the curved overlay plot and added to the writing. Both authors read and approved the final manuscript.

## Pre-publication history

The pre-publication history for this paper can be accessed here:


